# Association between genetic polymorphisms of interleukins and cerebral infarction risk: a meta-analysis

**DOI:** 10.1042/BSR20160226

**Published:** 2016-11-03

**Authors:** Jiantao Wang, Niannian Fan, Yili Deng, Jie Zhu, Jing Mei, Yao Chen, Heng Yang

**Affiliations:** * Department of Laboratory Medicine, Huaihe Hospital of Henan University, Kaifeng 475000, Henan, China; † Department of Cardiology, Third Affiliated Hospital of Third Military Medical University, Chongqing 400042, China; ‡ Department of Neurology, Third Affiliated Hospital of Third Military Medical University, Chongqing 400042, China; § Chongqing Medical University, Chongqing 400016, China

**Keywords:** cerebral infarction, interleukin, meta-analysis, polymorphism, risk

## Abstract

Interleukins (ILs) are the most typical inflammatory and immunoregulatory cytokines. Evidences have shown that polymorphisms in ILs are associated with cerebral infarction risk. However, the results remain inconclusive. The present study was to evaluate the role of ILs polymorphisms in cerebral infarction susceptibility. Relevant case-control studies published between January 2000 and December 2015 were searched and retrieved from the electronic databases of Web of Science, PubMed, Embase and the Chinese Biomedical Database. The odds ratio (OR) with its 95% confidence interval (CI) were employed to calculate the strength of association. A total of 55 articles including 12619 cerebral infarction patients and 14436 controls were screened out. Four ILs (IL-1, IL-6, IL-10 and IL-18) contained nine single nucleotide polymorphisms (SNPs; IL-1α −899C/T, IL-1β −511C/T and IL-1β +3953C/T; IL-6 −174G/C and −572C/G; IL-10 −819C/T and −1082A/G; IL-18 −607C/A and −137G/C). Our result showed that IL-1α −899C/T and IL-18 −607C/A (under all the genetic models), and IL-6 −572C/G (under the allelic model, heterogeneity model and dominant model) were associated with increased the risk of cerebral infarction (*P*<0.05). Subgroup analysis by ethnicity showed that IL-6 −174G/C polymorphism (under all the five models) and IL-10 −1082A/G polymorphism (under the allelic model and heterologous model) were significantly associated with increased the cerebral infarction risk in Asians. Other genetic polymorphisms were not related with cerebral infarction susceptibility under any genetic models. In conclusion, IL-1α −899C/T, IL-6 −572C/G and IL-18 −607C/A might be risk factors for cerebral infarction development. Further studies with well-designed and large sample size are still required.

## INTRODUCTION

Cerebral infarction (or ischaemic stroke), resulting from a blockage in the blood vessels supplying blood to the brain, or leakage outside the vessel walls, is the leading cause of acquired disability in adults and the second leading cause of dementia [1]. It constitutes the majority of cases of cerebrovascular accidents, and can be atherothrombotic or embolic [2]. According to the Oxford Community Stroke Project classification, cerebral infarction is classified as total anterior circulation infarct, partial anterior circulation infarct, lacunar infarct or posterior circulation infarct [3]. The incidence of cerebral infarction ranged from 210 to 600 per 100000 inhabitants per year according to the geographical difference [4,5]. Approximate 20% mortality is occurred at 1 month after the first stoke [5]. The risk factors are age, gender, tobacco smoking, hypertension, dyslipidaemia, diabetes and atrial fibrillation [6,7]. Increasing number of traditional risk factors was shown to be associated with long-term mortality in patients with cerebral infarction [8]. The symptoms of cerebral infarction are determined by the parts of the brain affected, and the pathology and pathophysiology of this disease are still not well understood [9]. Although many improvements such as surgical evacuation and thrombolytic drugs have been made for patients with cerebral infarction during the last decades, there is no specific treatment due to the severity of bleeding [10]. Preventing cerebral infarctions will be important in reducing the high morbidity and mortality rate [11]. Therefore, it is urgent to identify some important biomarkers to predict this disease and guide the treatment at its early onset.

Cerebral infarction is a complex multifactorial polygenic disease. It is well known that inflammation response affects brain tissue after a stroke, and cells and elements of the immune system are involved in all stages of ischaemic cascade [12]. Interleukins (ILs), a multifunctional group of immunomodulators that primarily mediate the leucocyte cross-talk, is critical to mounting any successful inflammation and immune responses [13]. There are 38 ILs so far, and they mainly regulate the immune cell proliferation, growth, differentiation, survival, activation and functions [14]. In addition, ILs are known to be involved in the pathogenesis of human inflammatory and autoimmune diseases [15,16]. Studies have shown that ILs are associated with atherosclerosis [17], and play an important role in cardiovascular disease [18–20]. ILs may be major players in the development and progression of cerebral infarction, and the detection of serum ILs might be helpful to assess the severity, therapeutic efficacy and prognosis of patients with cerebral infarction. The increasing of serum IL-6 levels may be related with the occurrence and development of acute cerebral infarction [21]. The lower serum IL-10 concentration was significantly associated with an increased likelihood of cerebral infarction [22,23]. The serum level of IL-18 was significantly elevated in the patients with acute cerebral infarction, and correlated with the volumes of infarction and the clinical neurologic impairment degree scores [24]. IL-33 was shown to be involved in the pathogenesis and/or progression of acute cerebral infarction [25]. Moreover, some specific ILs such as IL-6 might be an independently predictive biomarker for future mortality in the elderly after an ischaemic stroke [26].

Genetic polymorphisms of ILs may affect local serum levels of the proteins and reflect lifelong inflammation status. Recent data suggest that single nucleotide polymorphisms (SNPs) in ILs may contribute to modulating the effects of inflammatory cytokines on cerebral infarction [27]. Although many studies have identified the role of ILs polymorphisms in cerebral infarction risk, the results still remain inconclusive. For example, Rezk et al. [28] inferred that IL-1β −511C/T polymorphism might be associated with more severe functional and neurological impairments in patients with ischaemic stroke, whereas Zhang et al. [29] found no significant association between the IL-1β −511 C/T variant and ischaemic stroke. Therefore, we conducted this meta-analysis to review all the published articles on this issue and reevaluate the relationship between polymorphisms of ILs in cerebral infarction susceptibility to obtain a relatively reliable result.

## MATERIALS AND METHODS

### Literature search strategy

We performed a comprehensive literature search in the electronic databases of the Web of Science, PubMed, Embase and the Chinese Biomedical Database to retrieve relevant articles published between January 2000 and December 2015. The following MeSH terms: ‘cerebral infarction or brain infarction or cerebral ischaemic stroke’, ‘interleukin or IL or cytokine’, and ‘polymorphism or variant or mutation’ as well as their combinations were used as the searching keywords in conjunction with a highly sensitive search strategy. The references of retrieved articles were manually searched to obtain more related resources. Our study only focused on articles written in English and Chinese. When the same authors or laboratories published more than one articles in the same subjects, only the most recent full-text article was included.

### Inclusion and exclusion criteria

Eligible studies had to meet the following criteria: (1) case-control study evaluating the correlation of IL genetic polymorphisms in the pathogenesis of cerebral infarction; (2) the patients should be diagnosed by neuroimaging evidence with both CT and MRI, and meet the diagnostic criteria for cerebral infarction according to the World Health Organization's diagnostic criteria [30]; (3) the controls should be age-, sex-, ethnic-matched participants without other cardiovascular and cerebrovascular diseases and (4) the genotype information was available to be extracted, and the result was presented in odds ratio (OR) with its 95% confidence intervals (CI). The exclusion criteria were: (1) review reports or conference papers; (2) without control group; (3) with duplicated date and (4) studies not conducted in humans.

### Data extraction

According to the PRISMA guidelines, two of our authors assessed the quality of relevant articles independently. They should reach a final consensus on each item, and any disagreement was solved by discussed with the third author. The following information was extracted: the first author's name, published year, country, ethnicity, mean age, sample size, genotype frequencies, genotyping method and Hardy–Weinberg equilibrium (HWE) in controls.

### Statistical analysis

The relationship between IL genetic polymorphisms and cerebral infarction susceptibility was measured by the pooled OR and 95% CI. The *Z* test was used to estimate the statistical significance of pooled ORs (*P*-value less than 0.05 were considered statistically significant). For each genetic polymorphism, the allelic model, homologous model, heterogeneous model, dominant model and recessive model were calculated. Between-study heterogeneity was evaluated by the *Q*-statistic test and the *I*^2^ test. If the effect was homologous (the *Q*-test showed a *P* > 0.05 and *I*^2^ test exhibited <50%), the fixed-effect model was employed; otherwise, the random-effect model was used. All the statistical analysis was performed using the RevMan statistical software (version 5.3, the Cochrane Collaboration, Oxford, England).

## RESULTS

### Study characteristics

**Figure 1:**
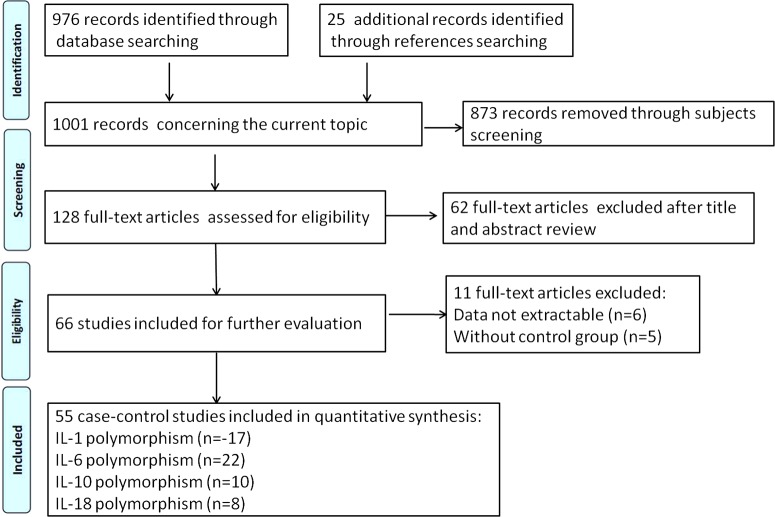
Flow chart of selection process in this meta-analysis

After applying the inclusion and exclusion criteria, we totally screened out 55 related articles, containing four genes (IL-1, IL-6, IL-10 and IL-18). Figure 1 presented the flow diagram of the selection of studies.

For IL-1, 17 articles contained three SNPs (IL-1α −899C/T, IL-1β −511C/T and IL-1β +3953C/T). Ten of them were conducted in Asian [29,31–39], six in Caucasian [40–45] and one in African [28]. All the genotype frequencies in controls followed the HWE.

For IL-6, 22 articles were included, containing two SNPs (−174G/C and −572C/G). Twelve (eight were written in Chinese [46–53] and four in English [54–57]) were conducted in Asian and 10 in Caucasian [40,58–66]. All the genotype frequencies in controls except the studies of Song et al., Li et al., Sun et al. and Tuttolomondo et al. were conformed to the HWE.

For IL-10, two polymorphisms (−819C/T and −1082A/G) from 10 articles (two were written in Chinese [67,68] and eight in English [61,69–75]) were included. Seven studies were conducted in Asians and three in Caucasians. The genotype distributions in all controls were consistent with HWE except the studies conducted by Zhang et al. and Marousi et al.

For IL-18, 8 articles (three in English [76–78] and five in Chinese [79–83]) contained 2 polymorphisms (−607C/A and −137G/C). All of them were conducted in Chinese population. The genotype distributions in all controls were consistent with HWE.

Table 1 listed the detailed characteristics of included studies. Table 2 exhibited the distribution information of genotypes in cerebral infarction cases and matched-controls.

**Table 1:** Main characteristics of included studies in this meta-analysis –, Not available; ARMS-PCR, amplification refractory mutation system PCR methods; PCR-RFLP, PCR-restriction fragment length polymorphism; PCR-SSP, PCR-sequence specific primer; RT-PCR, reverse transcription-PCR.

				Mean age		Sample size		
First author	Year	Country	Ethnicity	Cases	Controls	Cases	Controls	Genotyping methods
**IL-1**								
Seripa D	2003	Italy	Caucasian	65.8±10.4	63.7±14.0	101	110	PCR-RFLP
Um JY	2003	Korea	Asian	61.0±14.5	62.2±9.8	363	640	PCR-RFLP
Blading J	2004	Ireland	Caucasian	69 (35–99)	37.1 (18–65)	105	389	PCR-RFLP
Dziedzic T	2004	Poland	Caucasian	65.2±14.7	64.8±14.8	183	180	PCR-RFLP
lacoviello L	2005	Italy	Caucasian	35±7	35±8	134	134	PCR-RFLP
Rubattu S	2005	Italy	Caucasian	35.95±8.12	34.7±6.9	115	180	PCR-RFLP
Wei YS	2005	China	Asian	66.9±9.5	65.7±10.2	155	170	PCR-RFLP
Lai JT	2006	China	Asian	56.85±13.10	27.16±5.25	112	95	PCR-RFLP
Zhang GZ	2006	China	Asian	56±8	55±6	110	110	PCR-RFLP
Banerjee I	2008	India	Asian	58.6±14.2	57.4±8.8	112	212	PCR-RFLP
Zee RYL	2008	USA	Caucasian	62.1±0.5	61.7±0.5	258	258	PCR-RFLP
Dong RF	2009	China	Asian	60.31±10.51	58.77±10.83	82	82	PCR-RFLP
Li N	2010	China	Asian	63.88±7.36	62.87±7.57	371	371	PCR-RFLP
Ma XL	2012	China	Asian	46–75	44–70	65	130	PCR-RFLP
Zhao N	2012	China	Asian	59.2±10.71	62.32±10.68	1124	1163	PCR-RFLP
Zhang Z	2013	China	Asian	66.6±8.4	66.1±5.2	440	486	PCR-RFLP
Rezk NA	2015	Egypt	African	61.2±11.6	62.8±10.8	176	320	PCR-RFLP
**IL-6**								
Revilla M	2002	Spain	Caucasian	64.9±9.5	64.8±9.1	82	82	PCR-RFLP
Pola R	2003	Italy	Caucasian	76.8±8.4	76.2±7.1	119	133	PCR-RFLP
Blading J	2004	Ireland	Caucasian	69 (35–99)	37.1 (18–65)	105	389	PCR-RFLP
Flex A	2004	Italy	Caucasian	76.2±9.4	76.1±6.8	237	223	PCR-RFLP
Wei YS	2004	China	Asian	62.7±10.3	60.9±9.1	160	175	PCR-RFLP
Chamorro A	2005	Spain	Caucasian	67.0±10	64.0±10	273	105	PCR-RFLP
Song XJ	2005	China	Asian	68.23±9.58	66.08±8.62	66	98	PCR-RFLP
Lalouschek W	2006	Austria	Caucasian	53 (49–57)	49 (43–56)	404	415	PCR-RFLP
Li HJ	2006	China	Asian	64.92±11.16	63.91±11.96	112	105	PCR-RFLP
Yamada Y	2006	Japan	Asian	67.2±11.1	60.6±11.3	636	2010	PCR-SSP
Banerjee I	2008	India	Asian	58.6±14.2	57.4±8.8	112	212	PCR-RFLP
Liang J	2009	China	Asian	59.9±9.8	61.5±11.1	199	196	PCR-RFLP
Sun Y	2009	China	Asian	59.12±12.13	58.71±11.83	92	110	PCR-RFLP
Liu DF	2010	China	Asian	61.5±13.5	58.5±9.5	157	163	PCR-RFLP
Tong YQ	2010	China	Asian	61.52±9.68	60.61±9.11	748	748	Sequencing
Pan Y	2011	China	Asian	62.6±10.2	61.4 ±10.5	106	92	PCR-RFLP
Xiao H	2011	China	Asian	59.9±9.8	61.5 ±11.1	200	196	PCR-RFLP
Balcerzyk A	2012	Poland	Caucasian	8.75 (0.5–18)	7.5 (0.2–18)	80	138	PCR-RFLP
Chakraborty B	2012	India	Asian	54.0±10.9	52.5 ±9.8	100	120	PCR-RFLP
Tuttolomondo A	2012	Italy	Caucasian	71.9±9.75	71.4 ±7.45	96	48	PCR-RFLP
Xuan Y	2014	China	Asian	45.4±9.5	44.8±10.1	430	461	PCR-RFLP
Bazina A	2015	Croatia	Caucasian	54 (51–57)	55 (50–61)	114	187	RT-PCR
Ozkan A	2015	Turkey	Caucasian	63.57±15.3	62.29±12.6	42	48	RT-PCR
**IL-10**								
Zhang GZ	2007	China	Asian	55±9	35±5	204	131	PCR-RFLP
Munshi A	2010	India	Asian	49.3±17.34	47.01±16.78	480	470	ARMSPCR
Jin L	2011	China	Asian	–	–	189	92	PCR-RFLP
Marousi S	2011	Greece	Caucasian	68 (58–76)	69 (58–77)	145	145	RT-PCR
Sultana S	2011	India	Asian	53.72±11.11	54.06±10.98	238	226	ARMS PCR
Tuttolomondo A	2012	Italy	Caucasian	71.9±9.75	71.4±7.45	96	48	PCR-RFLP
He W	2015	China	Asian	–	–	260	260	PCR-RFLP
Jiang XH	2015	China	Asian	66.11±10.54	65.43±11.62	181	115	PCR-RFLP
Kumar P	2015	India	Asian	50.97±12.70	52.83±12.59	250	250	PCR-RFLP
Ozkan A	2015	Turkey	Caucasian	63.57±15.3	62.29±12.6	42	48	RT-PCR
**IL-18**								
Zhang N	2010	China	Asian	68.3±11.4	67.5±6.6	423	384	PCR-SSP
Li XQ	2011	China	Asian	62 (47–76)	59 (46–75)	98	100	PCR-SSP
Wang YJ	2011	China	Asian	64.2±13.1	63.9±12.9	218	218	PCR-SSP
Ren DL	2012	China	Asian	66.06±7.96	64.52±6.57	193	120	PCR-SSP
Lu JX	2013	China	Asian	65.7±8.8	64.6±9.9	386	364	PCR-RFLP
Wei GY	2013	China	Asian	58.5±12.1	59.6±12.8	153	114	PCR-RFLP
Dai XL	2014	China	Asian	63.88±7.36	62.87±7.57	371	371	PCR-RFLP
Shi JH	2015	China	Asian	62.4±9.3	61.8±10.6	322	322	PCR-RFLP

**Table 2:** Information of genotype distribution in cerebral infarction cases and controls among included studies in this meta-analysis

First author				Cases				Controls			HWE
**IL-1**											
IL-1α −899C/T	CC	CT	TT	C	T	CC	CT	TT	C	T	
Um JY	292	68	3	652	74	554	81	5	1189	91	0.57
Wei YS	115	37	3	267	43	146	23	1	315	25	0.99
Zhang GZ	84	23	3	191	29	97	13	0	207	13	0.80
Banerjee I	38	62	12	138	86	104	89	19	297	127	0.99
Dong RF	46	26	10	118	46	68	12	2	148	16	0.31
Li N	121	207	43	449	293	154	183	34	491	251	0.14
Zhao N	11	189	924	211	2037	10	220	933	240	2086	0.75
Zhang Z	145	232	63	522	335	200	237	49	637	358	0.22
Rezk NA	48	84	44	180	172	180	118	22	478	162	0.91
IL-1β −511C/T	CC	CT	TT	C	T	CC	CT	TT	C	T	
Seripa D	41	47	13	129	73	39	58	13	136	84	0.47
Dziedzic T	94	69	20	257	109	87	79	14	253	107	0.79
lacoviello L	66	59	9	191	77	52	61	21	165	103	0.91
Rubattu S	47	51	17	145	85	79	83	18	241	119	0.85
Lai JT	25	55	32	105	119	30	46	19	106	84	0.98
Zhang GZ	28	51	31	107	113	30	52	28	112	108	0.85
Zee RYL	113	123	22	349	167	111	120	27	342	174	0.81
Dong RF	52	23	7	127	37	46	26	10	118	46	0.15
Li N	93	170	108	356	386	101	178	92	380	362	0.74
Ma XL	42	17	6	101	29	87	39	4	213	47	0.99
Zhao N	298	561	265	1157	1091	323	583	257	1229	1097	0.98
Zhang Z	119	226	95	464	416	108	261	117	477	495	0.26
Rezk NA	53	87	36	193	159	206	101	13	513	127	0.99
IL-1β+3953C/T	CC	CT	TT	C	T	CC	CT	TT	C	T	
Um JY	332	30	1	694	32	593	46	1	1232	48	0.99
Blading J	66	35	4	167	43	240	125	24	605	173	0.38
Zhang GZ	97	13	0	207	13	106	4	0	216	4	0.98
Dong RF	52	24	6	128	36	57	20	5	134	30	0.25
Ma XL	34	19	12	87	43	82	42	8	206	58	0.71
**IL-6**											
−174G/C	GG	GC	CC	G	C	GG	GC	CC	G	C	
Revilla M	37	39	6	113	51	27	40	15	94	70	0.99
Pola R	56	48	15	160	78	28	58	47	114	152	0.45
Blading J	33	60	12	126	84	123	198	68	444	334	0.75
Flex A	100	115	22	315	159	66	99	68	231	235	0.07
Chamorro A	104	134	35	342	204	46	50	9	142	68	0.67
Song XJ	54	7	5	115	17	93	4	1	190	6	0.008
Lalouschek W	143	187	74	473	335	156	192	67	504	326	0.83
Li HJ	39	24	49	102	122	55	29	21	139	71	0.000
Banerjee I	77	35	0	189	35	156	52	4	364	60	0.99
Sun Y	32	20	40	84	100	59	28	23	146	74	0.000
Liu DF	138	19	0	295	19	153	10	0	316	10	0.92
Tong YQ	747	1	0	1495	1	743	5	0	1491	5	0.99
Balcerzyk A	21	43	16	85	75	40	76	22	156	120	0.37
Chakraborty B	57	35	8	149	51	73	39	8	185	55	0.68
Tuttolomondo A	40	46	10	126	66	14	33	1	61	35	0.003
Xuan Y	205	170	55	580	280	246	171	44	663	259	0.21
Bazina A	39	53	22	131	97	63	98	26	224	150	0.46
Ozkan A	4	22	16	30	54	14	21	13	49	47	0.69
−572C/G	CC	CG	GG	C	G	CC	CG	GG	C	G	
Wei YS	84	71	5	239	81	116	57	2	289	61	0.22
Yamada Y	412	199	25	1023	249	1138	760	112	3036	984	0.60
Liang J	103	89	7	295	103	127	66	3	320	72	0.23
Liu DF	34	33	3	101	36	51	24	5	126	34	0.65
Tong YQ	373	326	49	1072	424	424	267	57	1115	381	0.26
Pan Y	55	44	7	154	58	59	32	1	150	34	0.33
Xiao H	103	89	7	295	103	127	66	3	320	72	0.22
Xuan Y	267	127	35	661	197	318	122	21	758	164	0.12
**IL-10**											
−819C/T	CC	CT	TT	C	T	CC	CT	TT	C	T	
Zhang GZ	28	90	86	146	262	27	48	56	102	160	0.03
Jin L	12	82	95	106	272	7	37	48	51	133	0.99
Tuttolomondo A	63	14	19	140	52	26	17	5	69	27	0.69
He W	43	113	104	199	321	33	111	116	177	343	0.73
Jiang XH	32	73	76	137	225	18	44	53	80	150	0.24
−1082A/G	AA	AG	GG	A	G	AA	AG	GG	A	G	
Zhang GZ	202	2	0	406	2	120	11	0	251	11	0.88
Munshi A	92	241	147	425	535	63	218	189	344	596	0.99
Jin L	161	27	1	349	29	78	12	2	168	16	0.23
Marousi S	47	71	27	165	125	53	71	21	177	113	0.94
Sultana S	154	44	40	352	124	163	47	16	373	79	0.000
Tuttolomondo A	58	14	24	130	62	20	17	11	57	39	0.18
He W	41	124	95	206	314	29	108	123	166	354	0.77
Jiang XH	153	28	0	334	28	83	32	0	198	32	0.22
Kumar P	11	77	162	99	401	4	37	209	45	455	0.31
Ozkan A	11	26	5	48	36	19	18	11	56	40	0.28
**IL-18**											
−607C/A	CC	CA	AA	C	A	CC	CA	AA	C	A	
Zhang N	122	227	74	471	375	81	207	96	369	399	0.29
Li XQ	25	55	18	105	91	23	56	21	102	98	0.48
Ren DL	58	99	36	215	171	17	71	32	105	135	0.08
Lu JX	116	188	82	420	352	77	195	92	349	379	0.38
Dai XL	43	207	121	293	449	34	183	154	251	491	0.14
Shi JH	88	180	54	356	288	68	183	71	319	325	0.05
−137G/C	GG	GC	CC	G	C	GG	GC	CC	G	C	
Li XQ	76	19	3	171	25	62	33	5	157	43	0.98
Wang YJ	174	42	2	390	46	146	66	6	358	78	0.90
Ren DL	161	29	3	351	35	96	23	1	215	25	0.96
Wei GY	91	54	8	236	70	85	25	4	195	33	0.48
Dai XL	108	170	93	386	356	92	178	101	362	380	0.74
Shi JH	230	81	11	541	103	220	84	18	524	120	0.05

### Correlation between ILs polymorphisms and susceptibility to cerebral infarction

Table 3 showed the summary risk estimates for association between ILs polymorphisms and cerebral infarction.

**Table 3:** Meta-analysis on the association between ILs polymorphisms and cerebral infarction risk in total population *N*, number of included studies; Ph, *I*^2^, test of heterogeneity; F, fixed-effect model; R, random-effect model.

			Test of association		Test of heterogeneity		
SNPs	Comparisons	*N*	OR (95% CI)	*P*	Ph	*I*^2^	Model
**IL-1** IL-1α −899C/T	T versus C	9	1.69 (1.33, 2.14)	<0.0001	<0.0001	82%	R
	TT versus CC		2.32 (1.34, 3.99)	0.002	0.0007	70%	R
	CT versus CC		1.66 (1.44, 1.91)	<0.00001	0.07	45%	F
	TT + CT versus CC		1.89 (1.46, 2.44)	<0.00001	0.003	65%	R
	TT versus CT + CC		1.76 (1.18, 2.64)	0.006	0.0009	70%	R
IL-1β −511C/T	T versus C	13	1.11 (0.91, 1.35)	0.32	<0.0001	85%	R
	TT versus CC		1.27 (0.88, 1.84)	0.21	<0.0001	80%	R
	CT versus CC		1.04 (0.84, 1.29)	0.72	0.0001	69%	R
	TT + CT versus CC		1.09 (0.85, 1.40)	0.51	<0.0001	80%	R
	TT versus CT + CC		1.23 (0.93, 1.62)	0.14	<0.0001	71%	R
IL-1β +3953C/T	T versus C	5	1.24 (1.00, 1.54)	0.05	0.09	50%	F
	TT versus CC		1.47 (0.83, 2.60)	0.19	0.12	48%	F
	CT versus CC		1.21 (0.93, 1.57)	0.16	0.40	1%	F
	TT + CT versus CC		1.24 (0.97, 1.60)	0.09	0.29	20%	F
	TT versus CT + CC		1.43 (0.82, 2.51)	0.21	0.11	50%	F
**IL-6**							
−174G/C	C versus G	18	1.12 (0.88, 1.43)	0.37	<0.0001	86%	R
	CC versus GG		1.13 (0.68, 1.88)	0.64	<0.0001	85%	R
	GC versus GG		1.04 (0.92, 1.17)	0.56	0.02	47%	F
	CC + GC versus GG		1.09 (0.85, 1.41)	0.48	<0.0001	75%	R
	CC versus GC + GG		1.11 (0.71, 1.72)	0.65	<0.0001	83%	R
−572C/G	G versus C	8	1.31 (1.03, 1.66)	0.03	<0.0001	84%	R
	GG versus CC		1.48 (0.88, 2.48)	0.14	0.006	64%	R
	CG versus CC		1.38 (1.04, 1.83)	0.03	<0.0001	82%	R
	GG + CG versus CC		1.40 (1.05, 1.88)	0.02	<0.0001	84%	R
	GG versus CG + CC		1.28 (0.81, 2.02)	0.29	0.03	55%	R
**IL-10**							
−819C/T	T versus C	5	0.93 (0.80, 1.09)	0.38	0.64	0%	F
	TT versus CC		0.97 (0.71, 1.33)	0.86	0.34	12%	F
	CT versus CC		0.91 (0.54, 1.52)	0.71	0.03	62%	R
	TT + CT versus CC		0.93 (0.70, 1.22)	0.59	0.19	35%	F
	TT versus CT + CC		0.92 (0.75, 1.13)	0.42	0.56	0%	F
−1082A/G	G versus A	10	0.76 (0.57, 1.02)	0.07	<0.0001	82%	R
	GG versus AA		0.78 (0.46, 1.34)	0.37	0.0003	74%	R
	AG versus AA		0.76 (0.54, 1.07)	0.12	0.004	63%	R
	GG + AG versus AA		0.74 (0.52, 1.05)	0.09	0.0004	70%	R
	GG versus AG + AA		0.80 (0.51, 1.24)	0.31	<0.0001	80%	R
**IL-18**							
−607C/A	A versus C	6	0.76 (0.69, 0.84)	<0.00001	0.76	0%	F
	AA versus CC		0.56 (0.45, 0.68)	<0.00001	0.68	0%	F
	CA versus CC		0.71 (0.59, 0.84)	<0.0001	0.43	0%	F
	AA + CA versus CC		0.66 (0.55, 0.77)	<0.00001	0.48	1%	F
	AA versus CA + CC		0.70 (0.60, 0.82)	<0.0001	0.93	0%	F
−137G/C	C versus G	6	0.83 (0.62, 1.10)	0.20	0.003	72%	R
	CC versus GG		0.75 (0.55, 1.03)	0.08	0.43	0%	F
	GC versus GG		0.82 (0.57, 1.16)	0.26	0.005	70%	R
	CC + GC versus GG		0.81 (0.57, 1.14)	0.23	0.003	73%	R
	CC versus GC + GG		0.84 (0.64, 1.11)	0.21	0.58	0%	F

#### IL-1

**Figure 2:**
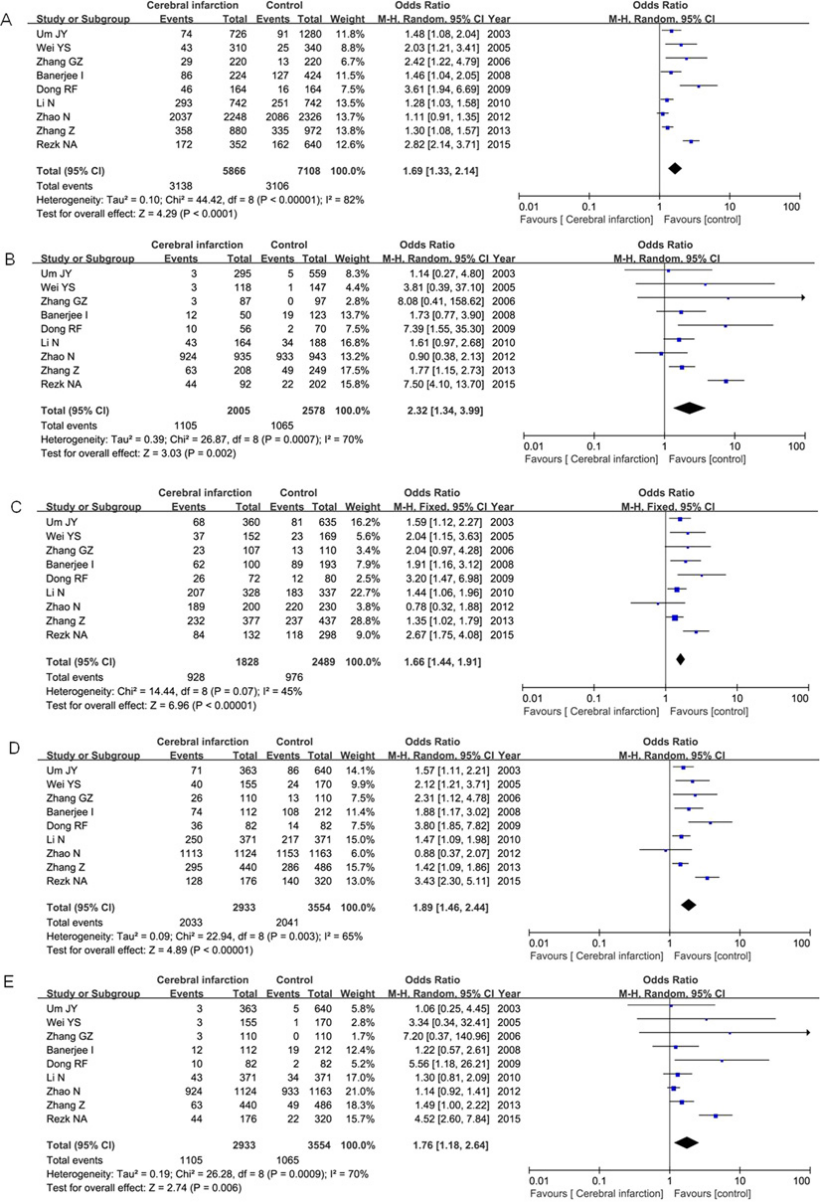
Meta-analysis of the relationship between the IL-1α −899C/T polymorphism and cerebral infarction risk under the allelic model (**A**), homologous model (**B**), heterogeneous model (**C**), dominant model (**D**) and recessive model (**E**).

For IL-1α −899C/T polymorphism, 9 articles included 2933 cerebral infarction patients and 3554 controls. The frequency of T allele was shown to be higher in cases than that in controls (53.5% versus 43.7%), and our result identified that IL-1α −899C/T polymorphism was associated with cerebral infarction risk under each genetic models (T versus C: OR=1.69, 95% CI=1.33–2.14, *P*<0.0001; TT versus CC: OR=2.32, 95% CI=1.34–3.99, *P*=0.002; CT versus CC: OR=1.66, 95% CI=1.44–1.91, *P*<0.00001; TT + CT versus CC: OR=1.89, 95% CI=1.46–2.44, *P*<0.00001; TT versus CT + CC: OR=1.76, 95% CI=1.18–2.64, *P*=0.006) as shown in Figure 2.

For IL-1β −511C/T polymorphism, there were 3271 cerebral infarction cases and 3619 controls from 13 articles. We did not detect a significant association between IL-1β −511C/T polymorphism and cerebral infarction susceptibility under any genetic models in the random-effect model (Table 3).

For IL-1β +3953C/T polymorphism, 5 articles contained 725 patients and 1353 controls. Our result found that there was no positive relationship between IL-1β +3953C/T polymorphism and cerebral infarction risk in the fixed-effect model as well (Table 3).

#### IL-6

**Figure 3:**
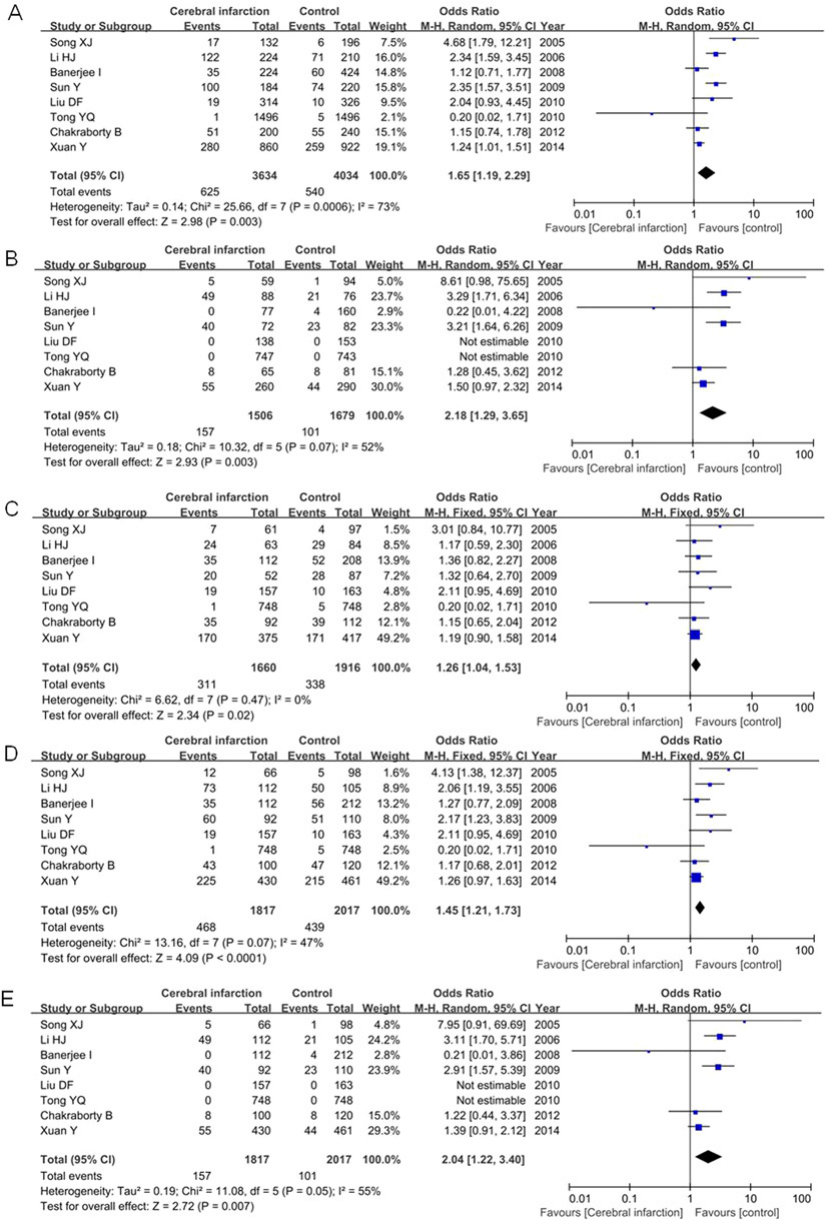
Forest plot of the relative strength of the association between IL-6 −174G/C polymorphism and cerebral infarction risk in Asians under the allelic model (**A**), homologous model (**B**), heterogeneous model (**C**), dominant model (**D**) and recessive model (**E**).

For IL-6 −174G/C polymorphism, 18 articles contained 3369 patients and 3795 controls. Our result did not find a significant relationship between IL-6 −174G/C polymorphism and cerebral infarction occurrence under any genetic models (Table 3). Subgroup analysis by ethnicity showed that this genetic variant was associated with increased the risk to cerebral infarction only in Asians (C versus G: OR=1.65, 95% CI=1.19–2.29, *P*=0.003; CC versus GG: OR=2.18, 95% CI=1.29–3.65, *P*=0.003; GC versus GG: OR=1.26, 95% CI=1.04–1.53, *P*=0.02; CC + GC versus GG: OR=1.45, 95% CI=1.21–1.73, *P*<0.0001; CC versus GC + GG: OR=2.04, 95% CI=1.22–3.40, *P*=0.007) as shown in Figure 3.

**Figure 4:**
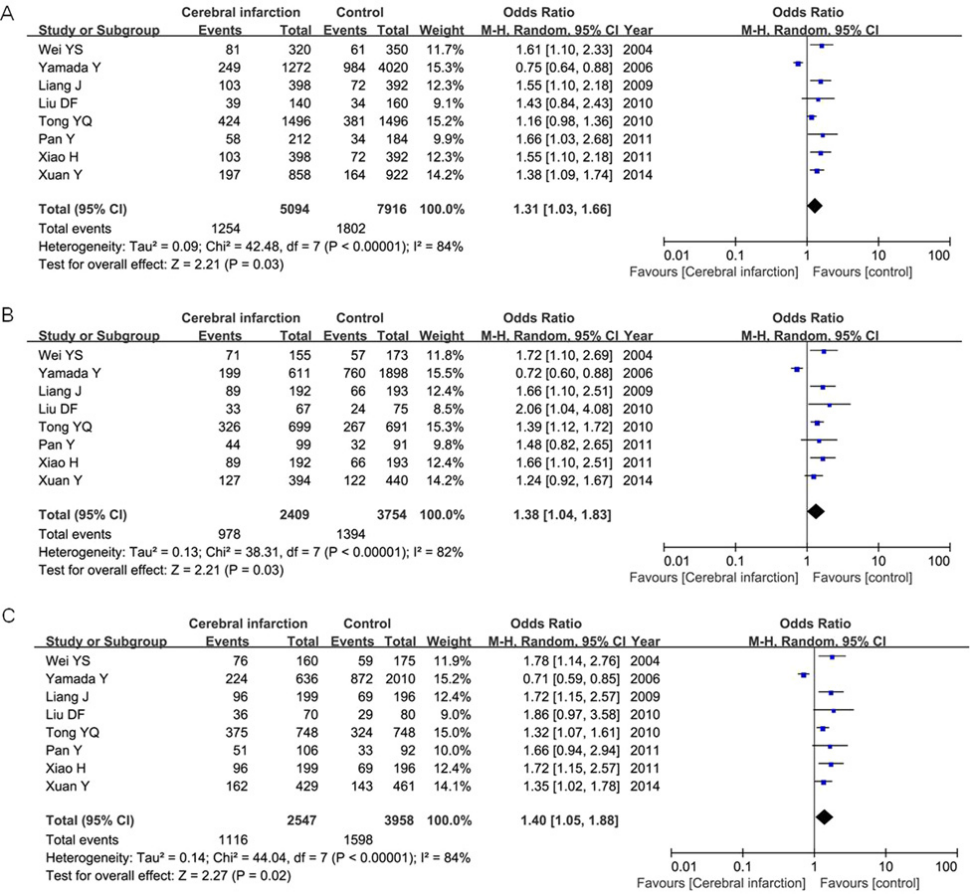
Meta-analysis of correlation of IL-6 −572C/G polymorphism in cerebral infarction risk under the allelic model (**A**: G versus **C**), heterogeneity model (**B**: CG versus CC) and dominant model (**C**: GG + CG versus CC) in the random-effect model.

For IL-6 −572C/G polymorphism, 8 articles contained 2547 patients and 3958 controls. Our result found that IL-6 −572C/G polymorphism was positively correlated with cerebral infarction risk under the allelic model (G versus C: OR=1.31, 95% CI=1.03–1.66, *P*=0.03), heterogeneity model (CG versus CC: OR =1.38, 95% CI=1.04–1.83, *P*=0.03) and dominant model (GG + CG versus CC: OR=1.40, 95% CI=1.05–1.88, *P*=0.02) in the random-effect model as shown in Figure 4.

#### IL-10

For IL-10 −819C/T mutation, 5 articles included 930 patients and 646 controls. Our result found no significant association between this genetic variant and cerebral infarction risk under any comparison models as shown in Table 3.

**Figure 5:**
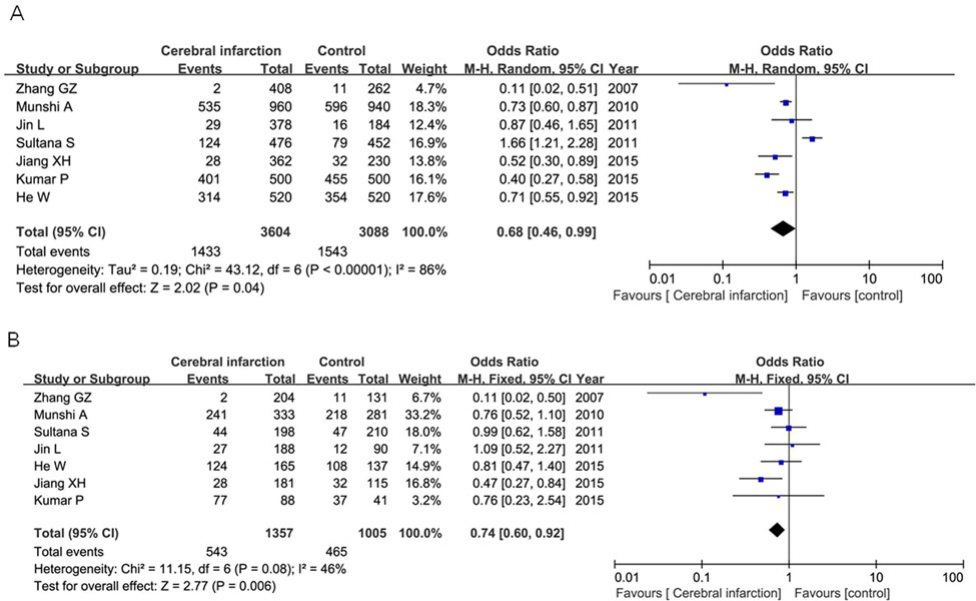
Forest plot of the association between IL-10 −1082A/G polymorphism and cerebral infarction risk under the allelic model (**A**) and heterologous model (**B**).

For IL-10 −1082A/G polymorphism, 2085 cases and 1785 controls from 10 relevant articles were screened out. This SNP was not associated with increased the susceptibility of cerebral infarction under each genetic models as well (Table 3). Subgroup analysis by ethnicity showed that IL-10 −1082A/G polymorphism was significantly associated with increased the cerebral infarction risk under the allelic model (OR=0.68, 95% CI=0.46–0.99, *P*=0.04) and heterologous model (OR=0.74, 95% CI=0.60–0.92, *P*=0.006) as shown in Figure 5.

#### IL-18

**Figure 6:**
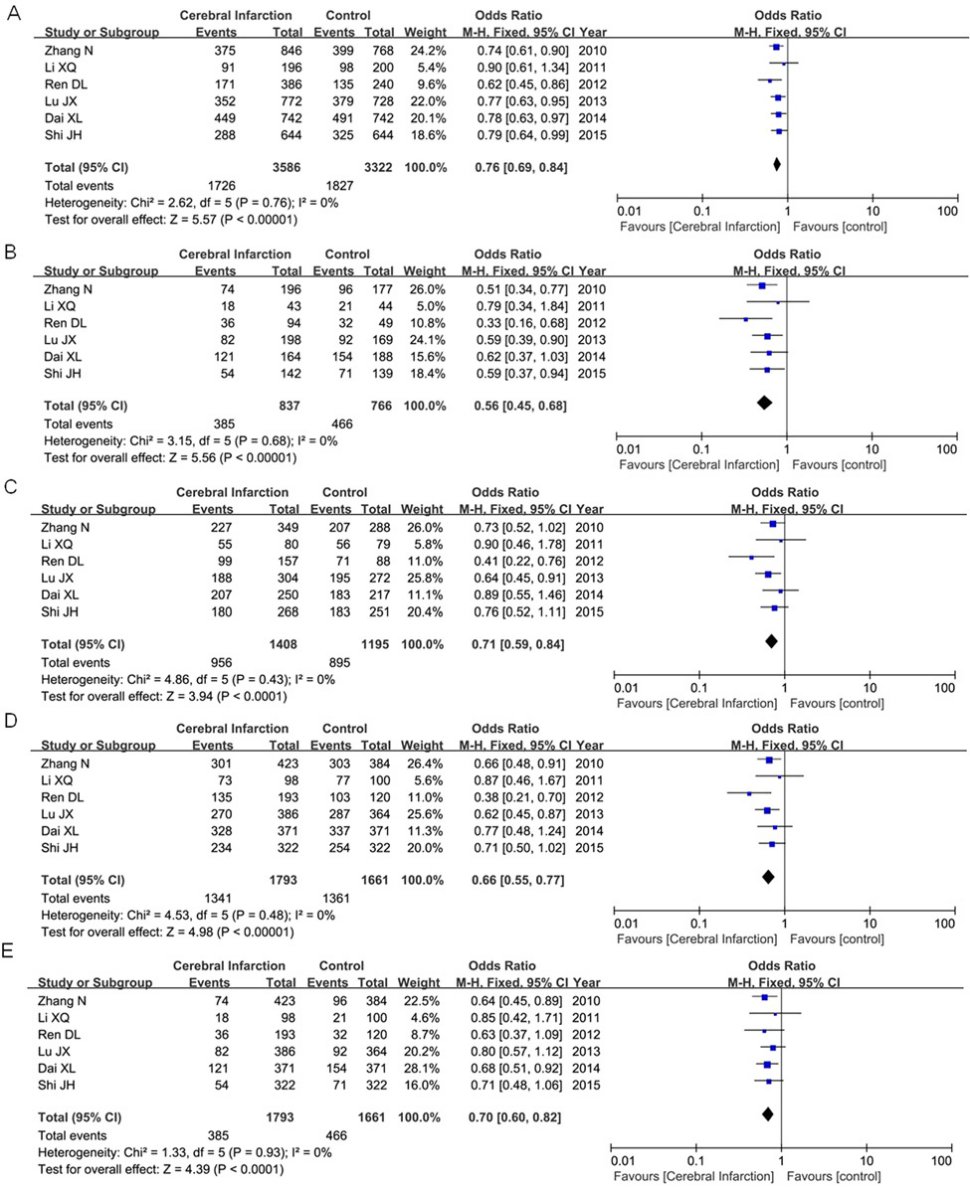
Forest plots for association between IL-18 −607C/A polymorphism and cerebral infarction risk under the allelic model (**A**), homologous model (**B**), heterogeneous model (**C**), dominant model (**D**) and recessive model (**E**).

For IL-18 −607C/A polymorphism, 6 articles contained 1793 cerebral infarction patients and 1661 healthy controls. No significant heterogeneity was detected, and the fixed-effect model was used. Our result found that the frequency of A allele was a little higher in controls than that in patients (55.0% versus 48.1%), but the A allele of IL-18 −607C/A polymorphism was associated with increased the risk of cerebral infarction (A versus C: OR=0.76, 95% CI=0.69–0.84, *P*<0.00001). This statistically significant was also observed in other genetic models (AA versus CC: OR=0.56, 95% CI=0.45–0.68, *P*<0.00001; CA versus CC: OR=0.71, 95% CI=0.59–0.84, *P*<0.0001; AA + CA versus CC: OR=0.66, 95% CI=0.55–0.77, *P*<0.00001; AA versus CA + CC: OR=0.70, 95% CI=0.60–0.82, *P*<0.0001). Figure 6 showed the result of IL-18 −607C/A polymorphism in cerebral infarction risk.

For IL-18 −137G/C polymorphism, five articles included 1355 cases and 1245 controls. Our result found that IL-18 −137G/C polymorphism was not associated with cerebral infarction risk under any genetic comparison models (Table 3).

### Sensitivity analysis and publication bias

**Figure 7:**
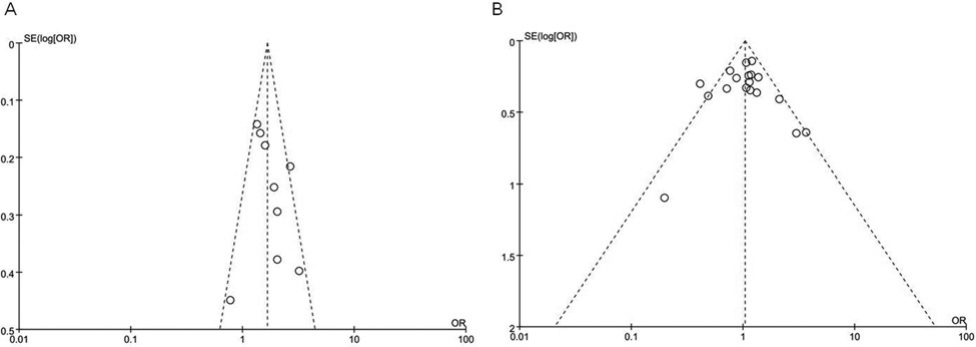
Funnel plot of IL-1α −899C/T (CT versus CC) and IL-6 −174G/C (GC versus GG) polymorphisms in cerebral infarction.

We successively omitted each single study respectively to confirm whether each included study affect the overall results. Our result found that the pooled ORs were not significantly changed. The funnel plots were used to evaluate the publication bias. All the plots were found to be roughly symmetrical, indicating no publication bias presented as shown in Figure 7. However, visual inspection of funnel plots did not guarantee that publication bias was absolutely absent.

## DISCUSSION

In this meta-analysis, we totally identified 55 relevant articles. Our results found that polymorphisms of IL-1α −899C/T and IL-18 −607C/A (under all the genetic models), and IL-6 −572C/G (under the allelic model, heterogeneity model and dominant model) were associated with increased the risk of cerebral infarction. Other genetic polymorphisms were not related with cerebral infarction susceptibility under any genetic models. Subgroup analysis by ethnicity showed that IL-6 −174G/C polymorphism (under all the five models) and IL-10 −1082A/G polymorphism (under the allelic model and heterologous model) were significantly associated with increased the cerebral infarction risk in Asians. This may be due to the higher frequency of C allele of IL-6 −174G/C and G allele of IL-10 −1082A/G in Asian populations. Our results were consistent with previous meta-analysis conducted by Jin et al. [84] and Yin et al. [85] which showed that IL-10 −1082 A/G polymorphism was associated with ischaemic stroke susceptibility in Asians, not consistent with the results from the studies of Kumar et al. [86] and Jin et al. [87] which showed that IL-6 −174G/C and −572C/G polymorphisms were not be associated with an increased susceptibility to ischaemic stroke, and Ye et al. [88] which inferred that IL-1β −511C/T polymorphism might be moderately associated with increased risk of ischaemic stroke.

Cerebral infarction is a complex vascular and metabolic process leading to neuronal death, and the loss of blood supply results in the death of that area of tissue [89]. The mechanisms for clinical deterioration in patients with ischaemic stroke are not completely understood. Interleukins are a kind of immunomodulating agents. They not only provide communication between immune cells, but also play a role in signalling the brain to produce neurochemical, neuroendocrine, neuroimmune and behavioural changes [90]. Several cytokines are released early after the onset of brain ischaemia, and studies have shown that IL-6 participated in the acute-phase response that follows focal cerebral ischaemia, and its levels on admission are associated with early clinical deterioration [91]. Furthermore, exploring these pathophysiological mechanisms underlying ischaemic tissue damage may direct rational drug design in the therapeutic treatment of stroke [92].

A growing body of evidence has indicated an important role of inflammatory cytokines in the pathogenesis of cerebral lesion following stroke [93]. They are critical to the pathogenesis of tissue damage in cerebral infarction [92]. IL-1 was shown to play a systemic inflammation role in acute brain injury [94]. Elevated IL-4 level in the human serum may be an important factor in cerebral infarction during the acute stage [95]. Increasing the serum IL-6 and IL-8 levels may be related with the occurrence and development of acute cerebral infarction [96]. Elevated IL-8 may contribute to stroke pathophysiology by activating polymorphonuclear leucocyte activation early after ischaemia [97]. IL-18 is involved in stroke-induced inflammation and that initial serum IL-18 levels may be predictive of stroke outcome [98].

Genetic polymorphisms may influence the expression level of ILs, which in turn may be associated with cerebral infarction. Analysis of genetic variation within genes coding for inflammatory mediators can offer some advantage compared with analyses of the plasma protein levels. Olsson et al. [99] showed a relationship between IL-1 receptor antagonist polymorphism and overall ischaemic stroke. Tong et al. [100] found that IL-4 variable number of tandem repeats polymorphism might influence the ischaemic stroke susceptibility in the Chinese Uyghur population. Luo et al. [101] demonstrated that the IL-8+781C/T polymorphism was associated with neurological recovery at the acute stage of atherosclerotic cerebral infarction in the Han Chinese population, and the patients with the CT genotype recovered better than those with other genotypes. Guo et al. [102] identified that genetic variation of rs4742 170 in IL33 is significantly associated with the developing of ischaemic stroke.

Several limitations were presented in this meta-analysis. Firstly, there was significant heterogeneity among included studies, which may affect the precision of outcome. Secondly, most of the included studies were conducted in Asian population, whereas other population should be included in the future analysis. Thirdly, due to lacking the detailed information, we could not perform a precise analysis by adjusting potentially suspected factors such as age, gender, smoking status and environmental factors. Lastly, the interaction of gene–gene and gene–environment should be considered.

In conclusions, our results suggested that polymorphisms of IL-1α −899C/T, IL-6 −572C/G and IL-18 −607C/A were positive correlated with increased the risk of cerebral infarction. Subgroup analysis by ethnicity showed that polymorphisms of IL-6 −174G/C and IL-10 −1082A/G were significantly associated with cerebral infarction risk in Asians. Future analysis with well-designed studies and large sample size are still needed to further investigate the association of polymorphisms in ILs and cerebral infarction.

## Abbreviations

CI: confidence interval
IL: interleukin
OR: odds ratio
SNP: single nucleotide polymorphism

## References

[1] Titov B., Matveeva N., Martynov M.Y., Favorova O. (2015). Ischemic stroke as a complex polygenic disease. Mol. Biol..

[2] Uchiyama S. (2006). [Cerebral infarction]. Nihon Rinsho.

[3] Amarenco P., Bogousslavsky J., Caplan L., Donnan G., Hennerici M. (2009). Classification of stroke subtypes. Cerebrovasc. Dis..

[4] Tsai C.-F., Thomas B., Sudlow C.L. (2013). Epidemiology of stroke and its subtypes in Chinese vs white populations: a systematic review. Neurology.

[5] Saliou G., Théaudin M., Join-Lambert Vincent C., Souillard-Scemama R. (2014). General Description of Cerebral Infarction. Practical Guide to Neurovascular Emergencies.

[6] Ihle-Hansen H. (2012). Risk factors for and incidence of subtypes of ischemic stroke. Funct. Neurol..

[7] Araki Y., Kumakura H., Kanai H., Kasama S., Sumino H., Ichikawa A., Ito T., Iwasaki T., Takayama Y., Ichikawa S. (2012). Prevalence and risk factors for cerebral infarction and carotid artery stenosis in peripheral arterial disease. Atherosclerosis.

[8] Gjerde G., Naess H. (2014). Risk factor burden predicts long-term mortality after cerebral infarction. Acta Neurol. Scand..

[9] Zülch K.-J. (2012). The Cerebral Infarct: Pathology, Pathogenesis, and Computed Tomography.

[10] Suzuki J. (2012). Treatment of Cerebral Infarction: Experimental and Clinical Study.

[11] Schut E.S., Lucas M.J., Brouwer M.C., Vergouwen M.D., van der Ende A., van de Beek D. (2012). Cerebral infarction in adults with bacterial meningitis. Neurocrit. Care.

[12] Siniscalchi A., Gallelli L., Malferrari G., Pirritano D., Serra R., Santangelo E., De Sarro G. (2014). Cerebral stroke injury: the role of cytokines and brain inflammation. J. Basic Clin. Physiol. Pharmacol..

[13] Fietta P., Costa E., Delsante G. (2013). Interleukins (ILs), a fascinating family of cytokines. Part I: ILs from IL-1 to IL-19. Theor. Biol. Forum.

[14] Chen Q., Carroll H.P., Gadina M. (2006). The newest interleukins: recent additions to the ever-growing cytokine family. Vitam. Horm..

[15] Yao X., Huang J., Zhong H., Shen N., Faggioni R., Fung M., Yao Y. (2014). Targeting interleukin-6 in inflammatory autoimmune diseases and cancers. Pharmacol. Ther..

[16] Tang C., Chen S., Qian H., Huang W. (2012). Interleukin-23: as a drug target for autoimmune inflammatory diseases. Immunology.

[17] Spears L.D., Razani B., Semenkovich C.F. (2013). Interleukins and atherosclerosis: a dysfunctional family grows. Cell Metab..

[18] Raman K., Chong M., Akhtar-Danesh G.-G., D'Mello M., Hasso R., Ross S., Xu F., Paré G. (2013). Genetic markers of inflammation and their role in cardiovascular disease. Can. J. Cardiol..

[19] Kernagis D.N., Laskowitz D.T. (2012). Evolving role of biomarkers in acute cerebrovascular disease. Ann. Neurol..

[20] Galea J., Brough D. (2013). The role of inflammation and interleukin-1 in acute cerebrovascular disease. J. Inflamm. Res..

[21] Juan C. (2013). Levels of serum C-reactive protein, tumor necrosis factor-α and interleukin-6 in patients with acute cerebral infarction. J. Xinxiang Med. Univ..

[22] Diao Z.-Y., Wang C.-L., Qi H.-S., Jia G.-Y., Yan C.-Z. (2015). Significance of decreased serum interleukin-10 levels in the progression of cerebral infarction. Clin. Exp. Med..

[23] Zhu Y., Yang H., Diao Z., Li Y., Yan C. (2016). Reduced serum level of interleukin-10 is associated with cerebral infarction: a case-control and meta-analysis study. Mol. Neurobiol..

[24] Huang Y., Su Z.-Q., Zhao Y. (2012). Clinical significance of serum IL-18 and MMP-9 levels in patients with acute cerebral infarction. J. Apoplexy Nervous Dis..

[25] Liu J., Xing Y., Gao Y., Zhou C. (2014). Changes in serum interleukin-33 levels in patients with acute cerebral infarction. J. Clin. Neurosci..

[26] Kwan J., Horsfield G., Bryant T., Gawne-Cain M., Durward G., Byrne C.D., Englyst N.A. (2013). IL-6 is a predictive biomarker for stroke associated infection and future mortality in the elderly after an ischemic stroke. Exp. Gerontol..

[27] Um J.Y., Jeong H.J., Park R.K., Hong S.H., Kim H.M. (2005). Aspects of gene polymorphisms in cerebral infarction: inflammatory cytokines. Cell Mol. Life Sci..

[28] Rezk N.A., Mohamad H.S. (2015). Influence of interleukin-1 gene cluster polymorphisms on the susceptibility and outcomes of acute stroke in egyptian patients. Cell Biochem. Biophys..

[29] Zhang Z., Liu L.-J., Zhang C., Yu Y.-P. (2013). Association between Interleukin-1 gene single nucleotide polymorphisms and ischemic stroke classified by TOAST criteria in the Han population of Northern China. BioMed. Res. Int..

[30] Tazaki Y. (1993). The diagnostic criteria of cerebral infarction and the differential diagnosis between cerebral infarction and cerebral hemorrhage. Nihon Rinsho.

[31] Um J.-Y., Moon K.-S., Lee K.-M., Kim H.-M. (2003). Interleukin-1 gene cluster polymorphisms in cerebral infarction. Cytokine.

[32] Lai J., Zhou D., Xia S., Shang Y., Zhu J., Pan J., Hua B., Zhu Y., Cui L. (2006). Association of interleukin-1 gene cluster polymorphisms with ischemic stroke in a Chinese population. Neurol. India.

[33] Zhang G., Pan S., Du R. (2006). The relationship between interleukin-1 gene polymorphisms and cerebral infarction. Chin. J. Cerebrovasc. Dis..

[34] Wei Y., Huang R., Lan Y. (2005). The association between interleukin-1 and interleukin-1 receptor antagonist polymorphisms and cerebral infarction. Chin. J. Gerontol..

[35] Banerjee I., Gupta V., Ahmed T., Faizaan M., Agarwal P., Ganesh S. (2008). Inflammatory system gene polymorphism and the risk of stroke: a case–control study in an Indian population. Brain Res. Bull..

[36] Ma X. (2012). Correlation analysis of IL-1 and TNF polymorphisms in cerebral infarction. Chin. J. Pract. Neurol. Dis..

[37] Dong R. (2009). Correlation Between Interleukin-1,4 Gene Polymorphism and Cerebral Infarction. Master's thesis.

[38] Zhao N. (2012). Association of Inflammatory Gene Polymorphisms with Ischemic Stroke in a Chinese Han Population. Master's thesis.

[39] Li N., He Z., Xu J., Liu F., Deng S., Zhang H. (2010). Association of PDE4D and IL-1 gene polymorphism with ischemic stroke in a Han Chinese population. Brain Res. Bull..

[40] Balding J., Livingstone W., Pittock S., Mynett-Johnson L., Ahern T., Hodgson A., Smith O.P. (2004). The IL-6 G-174C polymorphism may be associated with ischaemic stroke in patients without a history of hypertension. Ir. J. Med. Sci..

[41] Dziedzic T., Slowik A., Pera J., Szczudlik A. (2004). Lack of association between interleukin-1β polymorphism (−511) and ischaemic stroke. J. Neurol. Neurosurg. Psychiatry.

[42] Iacoviello L., Di Castelnuovo A., Gattone M., Pezzini A., Assanelli D., Lorenzet R., Del Zotto E., Colombo M., Napoleone E., Amore C. (2005). Polymorphisms of the interleukin-1β gene affect the risk of myocardial infarction and ischemic stroke at young age and the response of mononuclear cells to stimulation *in vitro*. Arterioscle. Thromb. Vasc. Biol..

[43] Seripa D., Dobrina A., Margaglione M., Matera M.G., Gravina C., Vecile E., Fazio V.M. (2003). Relevance of interleukin-1 receptor antagonist intron-2 polymorphism in ischemic stroke. Cerebrovasc. Dis..

[44] Rubattu S., Speranza R., Ferrari M., Evangelista A., Beccia M., Stanzione R., Assenza G.E., Volpe M., Rasura M. (2005). A role of TNF-α gene variant on juvenile ischemic stroke: a case–control study. Eur. J. Neurol..

[45] Zee R.Y., Hennessey H., Michaud S.E., Ridker P.M. (2008). Genetic variants within the interleukin-1 gene cluster, and risk of incident myocardial infarction, and ischemic stroke: a nested case-control approach. Atherosclerosis.

[46] Wei Y., Huang R., Zhang L. (2004). Association of IL-6 gene promoter −572C/G polymorphism and cerebral infarction. Youjiang Med. J..

[47] Song X. (2005). −174G/C Polymorphism of Lnterleukin-6 Gene and Ischemic Cerebrovaseular Disease. Master's thesis.

[48] Li H. (2006). Synergistic Effect of −174G/C Polymorphism of the Interleukin-6 Gene Promoter and 469E/K Polymorphism of the Intercellular Adhesion Molecule-1 Gene in Patients with Cerebral Infarction. Master's thesis.

[49] Yun S., Wu C., Deng H. (2009). Synergistic effect of-174 G/C polymorphism of the interleukin-6 gene promoter and 469 E/K polymorphism of the intercellular adhesion molecule-1 gene in the populations of the Han nationality in Shenzhen with cerebral infarction. Proc. Clin. Med..

[50] Liang J. (2009). The Relationship Between Interleukin-6 −572C/G, −597G/A Gene Polymorphism and Cerebral Infarction. Master's thesis.

[51] Liu D., Chen N., Wang D., Zhang B. (2010). Research on the polymorphism of IL-6 −174G/C gene of patients with cerebral infarction among Han people in Tangshan areas. Mod. Prev. Med..

[52] Pan Y., Wei Y., Huang J., Pan G. (2011). Association of interleukin-6 gene promoter −572C/G and −634C/G polymorphisms in patients with cerebral infarction. Chin. J. Lab. Diagn..

[53] Xiao H., Gu W., Ding Y. (2011). The study on the association between interleukin-6 −572C/G polymorphism and cerebral infarction. Chin. J. Nerv. Ment. Dis..

[54] Tong Y., Wang Z., Geng Y., Liu J., Zhang R., Lin Q., Li X., Huang D., Gao S., Hu D. (2010). The association of functional polymorphisms of IL-6 gene promoter with ischemic stroke: analysis in two Chinese populations. Biochem. Biophys. Res. Commun..

[55] Yang X., Feng L., Li C., Li Y. (2014). Association of IL-6 −174G>C and −572C>G polymorphisms with risk of young ischemic stroke patients. Gene.

[56] Chakraborty B., Chowdhury D., Vishnoi G., Goswami B., Kishore J., Agarwal S. (2013). Interleukin-6 gene-174 G/C promoter polymorphism predicts severity and outcome in acute ischemic stroke patients from north India. J. Stroke Cerebrovasc. Dis..

[57] Yamada Y., Metoki N., Yoshida H., Satoh K., Ichihara S., Kato K., Kameyama T., Yokoi K., Matsuo H., Segawa T. (2006). Genetic risk for ischemic and hemorrhagic stroke. Arterioscler. Thromb. Vasc. Biol..

[58] Pola R., Flex A., Gaetani E., Flore R., Serricchio M., Pola P. (2003). Synergistic effect of −174G/C polymorphism of the interleukin-6 gene promoter and 469 E/K polymorphism of the intercellular adhesion molecule-1 gene in Italian patients with history of ischemic stroke. Stroke.

[59] Revilla M., Obach M., Cervera V.C., Á., Dávalos A., Castillo J., Chamorro Á. (2002). A-174G/C polymorphism of the interleukin-6 gene in patients with lacunar infarction. Neurosci. Lett..

[60] Chamorro A., Revilla M., Obach V., Vargas M., Planas A. (2005). The −174G/C polymorphism of the interleukin 6 gene is a hallmark of lacunar stroke and not other ischemic stroke phenotypes. Cerebrovasc. Dis..

[61] Tuttolomondo A., Di Raimondo D., Forte G.I., Casuccio A., Vaccarino L., Scola L., Pecoraro R., Serio A., Clemente G., Arnao V. (2012). Single nucleotide polymorphisms (SNPs) of pro-inflammatory/anti-inflammatory and thrombotic/fibrinolytic genes in patients with acute ischemic stroke in relation to TOAST subtype. Cytokine.

[62] Balcerzyk A., Nowak M., Kopyta I., Emich-Widera E., Pilarska E., Pienczk-Ręcławowicz K., Kaciński M., Wendorff J., Zak I. (2012). Impact of the-174G/C interleukin-6 (IL-6) gene polymorphism on the risk of paediatric ischemic stroke, itssymptoms and outcome. Folia Neuropathol..

[63] Bazina A., Sertić J., Mišmaš A., Lovrić T., Poljaković Z., Miličić D. (2015). PPARγ and IL-6 −174G>C gene variants in Croatian patients with ischemic stroke. Gene.

[64] Ozkan A., Sılan F., Uludağ A., Degirmenci Y., Karaman H.I.O. (2015). Tumour necrosis factor alpha, ınterleukin 10 and ınterleukin 6 gene polymorphisms of ıschemic stroke patients ın south Marmara region of Turkey. Int. J. Clin. Exp. Pathol..

[65] Flex A., Gaetani E., Papaleo P., Straface G., Proia A.S., Pecorini G., Tondi P., Pola P., Pola R. (2004). Proinflammatory genetic profiles in subjects with history of ischemic stroke. Stroke.

[66] Lalouschek W., Schillinger M., Hsieh K., Endler G., Greisenegger S., Marculescu R., Lang W., Wagner O., Cheng S., Mannhalter C. (2006). Polymorphisms of the inflammatory system and risk of ischemic cerebrovascular events. Clin. Chem. Lab. Med..

[67] Jin L., Ni P., Wu J., Fu Y., Ge H. (2011). The correlation between gene polymorphism of IL-10-819 C/T and −1082 G/A and cerebral infarction. Lab. Med./Jianyan Yixue..

[68] Zhang G., Pan S., Du R., Lu B., Li W. (2007). The relationship between interleukin-10 gene polymorphisms and cerebral infarction. Chin. J. Cerebrovasc. Dis..

[69] Munshi A., Rajeshwar K., Kaul S., Al-Hazzani A., Alshatwi A.A., Babu M.S., Usha A., Jyothy A. (2010). Interleukin-10-1082 promoter polymorphism and ischemic stroke risk in a South Indian population. Cytokine.

[70] Sultana S., Kolla V.K., Jeedigunta Y., Penagaluru P.K., Joshi S., Rani P.U., Reddy P.P. (2011). Tumour necrosis factor alpha and interleukin 10 gene polymorphisms and the risk of ischemic stroke in south Indian population. J. Genet..

[71] Marousi S., Ellul J., Antonacopoulou A., Gogos C., Papathanasopoulos P., Karakantza M. (2011). Functional polymorphisms of interleukin 4 and interleukin 10 may predict evolution and functional outcome of an ischaemic stroke. Eur. J. Neurol..

[72] He W., Song H., Ding L., Li C., Dai L., Gao S. (2015). Association between IL-10 gene polymorphisms and the risk of ischemic stroke in a Chinese population. Int. J. Clin. Exp. Pathol..

[73] Jiang X.-H., Lin K.-X., Zhang Y.-X., Chen R.-H., Liu N. (2015). Correlating interleukin-10 promoter gene polymorphisms with human cerebral infarction onset. Neural. Regen. Res..

[74] Ozkan A., Sılan F., Uludağ A., Degirmenci Y., Karaman H.I.O. (2015). Tumour necrosis factor alpha, ınterleukin 10 and ınterleukin 6 gene polymorphisms of ıschemic stroke patients ın south Marmara region of Turkey. Int. J. Clin. Exp. Pathol..

[75] Kumar P., Kumar A., Sagar R., Misra S., Faruq M., Suroliya V., Vivekanandhan S., Srivastava A.K., Prasad K. (2015). Association between interleukin-10 −1082G/A gene polymorphism and risk of stroke in the north Indian population: a case-control study. J. Stroke Cerebrovasc. Dis..

[76] Zhang N., Yu J.-T., Yu N.-N., Lu R.-C., Ma T., Wang N.-D., Miao D., Song J.H., Tan L. (2010). Interleukin-18 promoter polymorphisms and risk of ischemic stroke. Brain Res. Bull..

[77] Lu J.-X., Lu Z.-Q., Zhang S.-L., Zhi J., Chen Z.-P., Wang W.-X. (2013). Correlation between interleukin-18 promoter-607C/A polymorphism and susceptibility to ischemic stroke. Braz. J. Med. Biol. Res..

[78] Shi J.-H., Niu L.-D., Chen X.-Y., Hou J.-Y., Yang P., Li G.-P. (2015). Investigation on the IL-18-607A/C and-137C/G on the susceptibility of ischemic stroke. Pakistan J. Med. Sci..

[79] Li X.-Q., Wu M.-H., Qin A.-L., Gao Y. (2011). Observation of IL-18 gene promoter 607C/A and 137G/C locus polymorphisms in acute cerebral infarction patients. Shandong Med. J..

[80] Ren D., Liu L., Song Y., Zhan J., Wang H., Zhang C. (2012). Relationship between interleukin-18 promoter polymorphism and ischemic stroke. Med. J. Qilu.

[81] Wei G., Chen J., Fu X., Li Z.-X., Jiang P.-P. (2013). Correlation between polymorphism of promoter region of interleukin-18 gene at position-137 G/C and acute cerebral infarction. Guangxi Med. J..

[82] Wang Y., Cheng Y. (2011). Correlations between interleukin 18 gene promoter-137 G/C polymorphism and plasma levels of interleukin-18 and acute cerebral infraction. Int. J. Cerebrovasc. Dis..

[83] Dai X., Wang Z. (2014). Correlation between interleukin-18 promoter-607C/A polymorphism and ischemic stroke risk. J. Hebei Med. Univ..

[84] Jin J., Li W., Peng L., Chen J., Li R., Wu P., Tan S. (2014). Relationship between interleukin-10-1082A/G polymorphism and risk of ischemic stroke: a meta-analysis. PLoS One.

[85] Yin G.-T., Ma Y.-T., Zheng Y.-Y., Yang Y.-N., Li X.-M., Fu Z.-Y., Zhang J.Z., Dai C.F., Liu F., Chen B.D. (2015). Polymorphisms of interleukin-10 genes on the risk of ischemic stroke in a meta-analysis. Int. J. Clin. Exp. Med..

[86] Kumar P., Yadav A.K., Kumar A., Sagar R., Pandit A.K., Prasad K. (2015). Association between Interleukin-6 (G174C and G572C) promoter gene polymorphisms and risk of ischaemic stroke: a meta-analysis. Ann. Neurosci..

[87] Jin X., Wang D., Zhou Y., Xiong H. (2014). Association between the interleukin-6-174 G/C polymorphism and risk of ischemic stroke: a meta-analysis. Genet. Mol. Res..

[88] Ye F., Jin X.-Q., Chen G.-H., Den X.-L., Zheng Y.-Q., Li C.-Y. (2012). Polymorphisms of interleukin-1 and interleukin-6 genes on the risk of ischemic stroke in a meta-analysis. Gene.

[89] Bounds J.V., Wiebers D.O., Whisnant J.P., Okazaki H. (1981). Mechanisms and timing of deaths from cerebral infarction. Stroke.

[90] Kronfol Z., Remick D.G. (2001). Cytokines and the brain: implications for clinical psychiatry. Am. J. Psychiatry.

[91] Vila N., Castillo, Dávalos J., Chamorro A., Á (2000). Proinflammatory cytokines and early neurological worsening in ischemic stroke. Stroke.

[92] Huang J., Upadhyay U.M., Tamargo R.J. (2006). Inflammation in stroke and focal cerebral ischemia. Surg. Neurol..

[93] Jin R., Liu L., Zhang S., Nanda A., Li G. (2013). Role of inflammation and its mediators in acute ischemic stroke. J. Cardiovasc. Transl. Res..

[94] Murray K.N., Parry-Jones A.R., Allan S.M. (2015). Interleukin-1 and acute brain injury. Front. Cell. Neurosci..

[95] Kim H.-M., Shin H.-Y., Jeong H.-J., An H.-J., Kim N.-S., Chae H.-J., Kim H.R., Song H.J., Kim K.Y., Baek S.H. (2000). Reduced IL-2 but elevated IL-4, IL-6, and IgE serum levels in patients with cerebral infarction during the acute stage. J. Mol. Neurosci..

[96] Han C.-P., Han Q.-W., Yao W.-J., Wang Q.-M. (2012). Levels of serum interleukin-6, interleukin-8 and tumor necrosis factor-α in patients with acute cerebral infarction. J. Xinxiang Med. Coll..

[97] Grau A.J., Reis A., Buggle F., Al-Khalaf A., Werle E., Valois N., Bertram M., Becher H., Grond-Ginsbach C. (2001). Monocyte function and plasma levels of interleukin-8 in acute ischemic stroke. J. Neurol. Sci..

[98] Zaremba J., Losy J. (2003). Interleukin-18 in acute ischaemic stroke patients. Neurol. Sci..

[99] Olsson S., Holmegaard L., Jood K., Sjögren M., Engström G., Lövkvist H., Blomstrand C., Norrving B., Melander O., Lindgren A., Jern C. (2012). Genetic variation within the interleukin-1 gene cluster and ischemic stroke. Stroke.

[100] Tong Y., Ye J., Wang Z., Zhang Y., Zhan F., Guan X., Geng Y.J., Hou S.Y., Li Y., Cheng J.Q. (2013). Association of variable number of tandem repeat polymorphism in the IL-4 gene with ischemic stroke in the Chinese Uyghur population. Genet. Mol. Res..

[101] Luo S. (2014). An association study between the +781C/T polymorphism in the interleukin-8 gene and the neurological recovery and prognosis of atherosclerotic cerebral infarctions. J. Neurophysiol. Neurol. Disord..

[102] Guo L., Zhou X., Guo X., Zhang X., Sun Y. (2013). Association of interleukin-33 gene single nucleotide polymorphisms with ischemic stroke in north Chinese population. BMC Med. Genet..

